# Replacing inorganic trace minerals with organic trace minerals with or without an exogenous amylase in the diet of finishing feedlot bulls: growth performance, carcass parameters, and rumen morphology

**DOI:** 10.1093/tas/txaf167

**Published:** 2025-12-18

**Authors:** D Polli, D D Millen, M B Gasparim, L T Feba, R C N Dinardi, G M Fogaça, G O Ribeiro, L J F Campos, L F Costa e Silva, M C S Pereira

**Affiliations:** College of Agricultural and Technological Sciences, São Paulo State University (UNESP), Dracena, SP, 17915-899, Brazil; Gasparim Animal Nutrition, Presidente Bernardes, SP, 19300-000, Brazil; School of Agricultural and Veterinary Sciences, São Paulo State University (UNESP), Jaboticabal, SP, 14884-900, Brazil; Gasparim Animal Nutrition, Presidente Bernardes, SP, 19300-000, Brazil; Gasparim Animal Nutrition, Presidente Bernardes, SP, 19300-000, Brazil; College of Agricultural and Technological Sciences, São Paulo State University (UNESP), Dracena, SP, 17915-899, Brazil; College of Agricultural and Technological Sciences, São Paulo State University (UNESP), Dracena, SP, 17915-899, Brazil; Department of Animal and Poultry Science, University of Saskatchewan, Saskatoon, SK, S7N 5A8, Canada; Alltech do Brasil Agroindustrial LTDA, Maringá, PR, 87030-405, Brazil; Alltech do Brasil Agroindustrial LTDA, Maringá, PR, 87030-405, Brazil; College of Agricultural and Technological Sciences, São Paulo State University (UNESP), Dracena, SP, 17915-899, Brazil

**Keywords:** enzyme, finishing, papillae, proteinate, zebu

## Abstract

This study assessed the growth, carcass traits, and rumen morphology of feedlot cattle fed reduced levels of organic trace minerals (OTM) in proteinate forms and Se-yeast, replacing inorganic trace minerals (ITM) sources in combination with exogenous amylase. One hundred and twenty commercial yearling Nellore bulls with an initial body weight (BW) of 349.20 ± 22.90 kg were allocated to 24 pens. The study employed a completely randomized block design with a 2 × 2 factorial treatment arrangement. Factors included ITM or OTM sources/level with or without exogenous amylase (Amaize, Alltech, Maringá, PR, Brazil [0.5 g/kg of dry matter]). The ITM supplement contained Co, Cu, Fe, Mn, and Zn in sulfate form, with Se provided as sodium selenite. The OTM supplement provided Co, Cu, Fe, Mn, and Zn in proteinate form at 50% of the levels supplied by the ITM supplement, while Se, as Se-yeast, was included at the same level as in the ITM supplement. Both supplements also contained Cr as Cr-yeast and I as sodium iodide. Cattle were fed the same diets, except for the inclusion of trace minerals and exogenous amylase. There was no interaction (*P ≥* 0.12) between trace mineral and exogenous amylase for any variable evaluated. Cattle fed OTM tended to have higher average daily gain (ADG; *P = *0.07) and gain-to-feed (G: F) ratio (*P = *0.06) compared to cattle fed ITM. Cattle fed exogenous amylase had greater dry matter intake (DMI), whether measured in kg/d (*P = *0.03) or as a percentage of BW (*P = *0.02), with no (*P ≥*0.10) effect on ADG and G: F ratio compared to cattle not fed exogenous amylase. There was no main effect of trace mineral or exogenous amylase inclusion (*P ≥* 0.15) on any of the carcass traits evaluated, except for final Biceps femoris fat thickness (*P = *0.05), which were higher in cattle fed OTM. Rumenitis score and rumen morphology were also not different (*P ≥*0.14). Overall, feeding reduced OTM levels did not exert adverse effects on growth, carcass traits, or rumen morphology in feedlot Nellore cattle, while modestly enhancing ADG, G: F ratio, and Biceps femoris fat thickness compared to ITM sources. The exogenous amylase inclusion increased DMI without differences in ADG, G: F ratio, carcass characteristics, or rumen morphology.

## Teaser

Modest benefits to feedlot growth efficiency were observed in Nellore cattle when reduced levels of organic trace minerals were used in place of inorganic sources, while higher feed intake occurred with exogenous amylase without affecting carcass traits.

## Introduction

Trace minerals serve as indispensable cofactors for numerous metalloenzymes involved in regulating oxidative balance, nutrient utilization, and cellular processes such as proliferation and programmed cell death (NASEM 2016; Goff 2018). Because most conventional feed ingredients contain inadequate amounts of trace minerals, dietary supplementation becomes necessary. Traditionally, inorganic trace minerals (ITM) have been the primary choice in animal nutrition, most commonly provided in the form of inorganic salts such as oxides, chlorides, sulfates, and carbonates (Hilal et al. 2016).

In general, ITM sources tend to show lower bioavailability and stability than organic trace mineral (OTM) sources, including amino acid chelates, proteinates, and mineral complexes, which are absorbed and less susceptible to antagonistic interactions with other dietary components in the gastrointestinal tract (Spears 1996, 2003). The OTM sources also may help mitigate nutrient antagonisms in the gut and may improve mineral bioavailability (Brown and Zeringue 1994; Goff 2018). According to Goff (2018), OTM sources may support mineral requirements at lower inclusion levels than inorganic forms due to their greater absorption efficiency in ruminants.

Over the past decade, grain inclusion levels in Brazilian finishing diets have increased (Millen et al. 2009; Monsalve and Millen 2025), with flint corn remaining the predominant grain used in feedlots (Monsalve and Millen 2025). Flint corn contains a higher proportion of vitreous endosperm than dent corn (Philippeau et al. 1999), a characteristic associated with reduced ruminal starch digestibility (Philippeau and Michalet-Doreau 1998) and throughout the digestive tract (Corona et al. 2006). Although grain processing remains the primary strategy to enhance starch utilization and improve cattle performance (Zinn et al. 2002), the inclusion of exogenous amylase in finishing diets has been proposed as an additional approach to support starch digestion in both dairy (Gencoglu et al. 2010) and beef (Tricarico et al. 2007) cattle.

Research evaluating OTM and ITM supplementation in feedlot cattle has reported variable effects on growth performance, ruminal morphology, mineral status, and metabolic responses (Spears 1996; Spears 2003; Caldera et al. 2017; Heldt and Davis 2019; Lippy et al. 2022; Guimaraes et al. 2022; Loh et al. 2025), suggesting that OTM responses depend on mineral form, dose, diet composition, stress level, and the physiological state of the animal. Although OTM supplementation is not expected to directly modify starch digestion, improved mineral status may affect metabolic pathways related to antioxidant capacity, epithelial function, or microbial activity, which could indirectly influence nutrient use in high-grain diets. Given the increasing use of grain-based diets in Brazil, understanding how reduced OTM inclusion levels interact with finishing diets remains relevant for optimizing feedlot performance. Based on that, we hypothesized that feeding OTM to finishing cattle, either alone or in combination with exogenous amylase, would improve growth, carcass characteristics, and help maintain rumen health. To test this hypothesis, this study evaluated the growth, carcass traits, and rumen morphology of feedlot Nellore cattle when ITM were replaced with reduced levels of OTM, in proteinate forms and Se-yeast, in combination with exogenous amylase.

## Materials and methods

All procedures involving animals in this study were approved by the São Paulo State University Animal Research Ethics Committee (protocol number: 21/2022).

### Experimental design and treatments

The study was conducted at Gasparim Animal Nutrition Feedlot Experimental Center (Presidente Bernardes, SP, Brazil). One hundred and twenty commercial yearling Nellore bulls were purchased from a local farm, with an initial body weight (BW) of 349 ± 22.9 kg. They were allocated to 24 pens (6 × 17 m; 1 m of bunk space per animal; 5 animals per pen). The study used a completely randomized block design (initial BW as the blocking criteria) with a 2 × 2 factorial arrangement. Factors included ITM or OTM sources/levels with or without an exogenous amylase (Amaize, Alltech, Maringá, PR, Brazil; 0.5 g/kg of dry matter [DM]). The ITM supplement contained Co, Cu, Fe, Mn, and Zn in sulfate form, with Se provided as sodium selenite to meet NASEM (2016) recommendation. The OTM supplement provided Co, Cu, Fe, Mn, and Zn in proteinate form at 50% of the levels supplied by the ITM supplement, while Se was included as Se-yeast at the same level as in the ITM supplement. Both supplements also contained Cr as Cr-yeast and I as sodium iodide, included at the same level.

### Animal care and feeding description

Before the start of the study, all animals were dewormed (4 mL/40 kg of BW; Parasules 10, Microsules, Porto Alegre, RS, Brazil) and vaccinated against *Clostridium* (5 mL subcutaneous; Cattlemaster Gold, Zoetis, São Paulo, SP, Brazil) and respiratory diseases (5 mL subcutaneous; StarVac, Labovet, Brazil). The ITM and OTM supplement (Alltech, Maringá, PR, Brazil) were offered as a top-dressing and contained (DM basis, g/kg): Ca: 218; P: 20; S: 25; Mg: 35; K: 40; Na: 70, all provided in inorganic form, sodium monensin: 900 mg/kg; Vitamin A 60000 IU/Kg; Vitamin D 8000 IU/kg; Vitamin E 500 IU/kg. The ITM supplement contained (DM basis, mg/kg): Co: 30; Cr: 15; Cu: 450; Mn: 850; Se: 5; Zn: 1350. The Co, Cu, Mn, and Zn were offered in sulfate form, with Se provided as sodium selenite. The OTM supplement contained (DM basis, mg/kg): Co: 15; Cr: 15; Cu: 225; 100; Mn: 425; Se: 5; Zn: 675. The Co, Cu, Mn, and Zn were offered as proteinates, and Se as Se-yeast. Both supplements also contained Cr as Cr-yeast and I as sodium iodide. Exogenous amylase (Amaize, Alltech, Maringá, PR, Brazil) was included at 0.5 g/kg of DM. A mycotoxin adsorbent (Mycosorb, Alltech, Maringá, PR, Brazil) was included in both supplements at 0.6 g/kg of DM.

Cattle were fed identical diets that differed only in the sources and levels of trace minerals and exogenous amylase. The animals were fed twice daily at 0800 (50% of the total ration) and 1500 (50% of the total ration). Feed delivery was adjusted daily for each pen to maintain refusals between 1% and 3%, thereby ensuring ad libitum intake, with unrestricted access to the water trough. The study lasted 111 days, including a 26-day adaptation phase followed by 85 days on a finishing diet. The step-up adaptation protocol consisted of ad libitum intake with progressively higher proportions of concentrate ingredients until the cattle reached the level required for the finishing diet. The adaptation period extended over 26 days, during which two transitional diets containing 75% (Step 1) and 80% (Step 2) concentrate were provided for 15 and 11 days, respectively. Experimental diets (Table 1) were formulated using the Beef Cattle Nutrient Requirements Model (BCNRM, 2016) to meet the recommendations for growing and finishing cattle, and they consisted of *Panicum maximum* cv. Mombaça silage, peanut hulls, finely ground corn, high-moisture re-hydrated corn grain, dried distillers’ grains, soybean hulls, cottonseed meal, rumen-protected fat, urea, and mineral/vitamin supplement. The analyzed mineral composition of the experimental diets is presented in Table 2.

**Table 1.** Ingredients and chemical composition of experimental diets.

Item	Diets^a^		
	Step 1	Step 2	Finishing
***Ingredients, % of DM^b^***			
*** Panicum maximum* cv. Mombaça silage**	15.00	10.00	5.00
*** *Peanut hulls**	10.00	10.00	10.00
*** *Corn grain fine grind**	12.00	16.00	20.00
*** *High-moisture re-hydrated corn grain**	12.00	16.00	20.00
*** *Dried distillers grains**	19.00	19.00	19.00
*** *Soybean hulls**	18.00	18.00	18.00
*** *Cottonseed meal**	11.25	7.75	4.30
*** *Rumen-protected fat**	0.50	0.75	1.00
*** *Urea**	0.20	0.20	0.20
*** *Supplement**	2.05	2.30	2.50
***Nutrient content^c^, % of DM***			
*** *DM, %**	65.69 ± 0.38	67.24 ± 0.38	69.11 ± 0.21
*** *Crude protein**	20.57 ± 0.62	17.80 ± 0.32	16.81 ± 0.37
*** *Neutral detergent fiber**	38.40 ± 0.70	37.42 ± 2.30	29.79 ± 2.48
*** *Starch**	17.04 ± 1.30	25.70 ± 31.92	28.71 ± 1.21
*** *Ether extract**	3.00 ± 0.48	2.29 ± 0.19	2.95 ± 0.20
*** *NEm^d^, Mcal/kg**	1.65 ± 0.03	1.62 ± 0.06	1.79 ± 0.01
*** *NEg^e^, Mcal/kg**	1.20 ± 0.02	1.18 ± 0.05	1.31 ± 0.01

a Cattle were fed the same diets, except for the trace mineral and exogenous amylase inclusion.

b Dry matter.

c Nutrient content is expressed as means ± SD (*n* = 3).

d Net energy for maintenance was calculated from feed samples using the NASEM (2016) equations.

e Net energy for gain was calculated from feed samples using the NASEM (2016) equations.

**Table 2.** Analyzed^a^ Mineral composition of the experimental diets.

Item	Diets^b^					
	Step 1		Step 2		Finishing	
	Inorganic	Organic	Inorganic	Organic	Inorganic	Organic
***Macro-minerals, g/kg***						
** Ca**	7.17 ± 0.69	6.17 ± 0.90	6.33 ± 0.75	6.33 ± 1.37	6.67 ± 0.75	7.17 ± 0.69
** K**	11.33 ± 0.47	10.33 ± 1.37	10.17 ± 0.69	9.83 ± 1.34	8.67 ± 0.75	9.33 ± 0.75
** Mg**	4.17 ± 0.37	4.33 ± 0.47	3.67 ± 0.47	3.83 ± 0.69	3.17 ± 0.37	3.83 ± 0.37
** Na**	0.95 ± 0.08	0.62 ± 0.25	0.85 ± 0.16	0.62 ± 0.23	0.87 ± 0.14	0.88 ± 0.09
** P**	6.50 ± 0.50	5.33 ± 0.94	6.33 ± 0.47	5.33 ± 0.94	6.00 ± 0.58	5.50 ± 0.76
***Trace minerals, mg/kg***						
** Co**	8.80 ± 1.17	<5.00 ± 0.00	9.00 ± 1.15	<5.00 ± 0.00	8.50 ± 2.14	<5.00 ± 0.00
** Cu**	17.33 ± 1.70	13.20 ± 1.94	20.67 ± 1.70	12.50 ± 0.76	16.00 ± 0.01	11.17 ± 0.37
** Mn**	43.00 ± 1.63	38.67 ± 1.25	40.67 ± 1.70	33.17 ± 1.34	34.83 ± 1.46	27.00 ± 1.00
** Zn**	117.00 ± 1.00	77.25 ± 2.17	102.80 ± 0.75	79.60 ± 1.36	111.67 ± 1.36	78.40 ± 1.00

a Macro an trace minerals were determined by flame atomic absorption spectrophotometry (240FS, Agilent Technologies, Santa Clara, CA, USA) according to AOAC (1999; method 968.08). Phosphorus concentrations were quantified using a molecular absorption spectrophotometer (700 plus, FEMTO, São Paulo, SP, Brazil) described in AOAC (1999; method 965.17).

b Nutrient content is expressed as means ±SD (*n* = 3).

Feed bunks were cleaned daily at 0700 throughout the experimental period, before the morning feeding. Feed ingredient samples were collected weekly and analyzed for DM content (AOAC 1990; method 930.15). The inclusion rates of dietary ingredients were then adjusted according to the most recent DM values. Feed ingredients and refusals were not analyzed for sorting index. Dried samples of each ingredient were pooled into three composite samples and ground in a hammer mill (MA340, Marconi, Piracicaba, SP, Brazil) to pass through a 1 mm sieve. All samples were subsequently analyzed for crude protein (AOAC 1997; method 990.02). Neutral detergent fiber was determined using a fiber analyzer (model A200; ANKOM Technology Corp., Fairport, NY) with the addition of α-amylase, as described by Van Soest et al. (1991). Starch concentration was measured according to Pereira and Rossi (1995), following prior removal of soluble carbohydrates as described by Hendrix (1993). Ether extract content was determined according to AOAC (1990; method 920.39). Net energy requirements for maintenance and gain were calculated based on feed composition using the equations provided by NASEM (2016). Concentrations of Ca, K, Mg, Na, Co, Cu, Mn, and Zn in feed samples were determined by flame atomic absorption spectrophotometry (240FS, Agilent Technologies, Santa Clara, CA, USA) according to AOAC (1999; method 968.08). Phosphorus concentrations were quantified using a molecular absorption spectrophotometer (700 plus, FEMTO, São Paulo, SP, Brazil) described in AOAC (1999; method 965.17).

### Performance and carcass traits

At the start and end of the study, individual bulls were weighed after 16 h of feed withdrawal. Daily dry matter intake (DMI) was determined by weighing the feed DM offered and subtracting the amount of DM refused, with values expressed both in kg and as a percentage of BW. The average daily gain (ADG) was calculated from the BW records as the difference between final and initial BW divided by the number of feeding days. The gain-to-feed ratio (G: F) was calculated as the ratio of ADG to DMI. Ultrasound assessments were conducted on days 1 and 111 of the study period using an Aloka SSD-1100 Flexus RTU unit (Aloka Co. Ltd, Tokyo, Japan) equipped with a 17.2-cm, 3.5-MHz transducer. Measurements included 12th-rib fat thickness, Biceps femoris fat thickness, longissimus muscle area, and marbling score to evaluate carcass fat and muscle deposition throughout the experimental period, following the methodology described by Perkins et al. (1992). All ultrasound images were evaluated by a trained technician using the Bovine Image Analysis software (Designer Genes Technologies, Presidente Prudente, SP, Brazil). At the end of the trial, cattle were transported approximately 160 km to a federally inspected slaughterhouse (JBS S.A., Andradina, SP, Brazil) for slaughter. Hot carcass weight (HCW) was obtained after the kidney and pelvic fat removal, and dressing percentage was calculated as the ratio of HCW to final BW.

### Rumenitis score, rumen morphology, and liver abscess

Following evisceration, rumenitis was evaluated and each cleaned rumen received a score based on the lesion classification proposed by Bigham and McManus (1975). Scores ranged from 0, indicating an intact epithelium with no visible lesions, to 10, representing severe ulcerative damage. Two trained evaluators, blinded to treatment allocation, independently scored each rumen, and the average of their assessments was used for statistical analysis. A tissue sample was then collected from the cranial sac of each rumen, rinsed in phosphate-buffered saline, and prepared for morphometric assessment according to the procedures of Resende Júnior et al. (2006). Liver abscesses were also recorded and classified by incidence following the methodology described by Brink et al. (1990).

### Statistical analysis

Data were analyzed using PROC MIXED of SAS (SAS Inst., Inc., Cary, NC). The pen was considered the experimental unit (*n = *6 per treatment). The model included fixed effects of trace mineral source (ITM *vs*. OTM), exogenous amylase inclusion (with *vs*. without), and their interaction, with initial BW blocks treated as random effects (block 1: 310.91 kg; block 2: 329.92 kg; block 3: 338.19 kg; block 4: 350.76 kg; block 5: 368.60 kg; block 6: 396.85 kg). Data were checked for normality (Shapiro–Wilk and Kolmogorov–Smirnov tests) and variance homogeneity (SAS GROUP option) before analysis. Treatment means were compared using Tukey’s test. Significance was considered at *P ≤* 0.05, and trends at 0.05 < *P <* 0.10.

## Results and discussion

There were no interactions (*P ≥* 0.12; Table 3) between trace mineral and exogenous amylase inclusion for any variable evaluated in this study. Since no interactions were found, the discussion will focus on the individual effects of mineral source and exogenous amylase. This study hypothesized that enhanced feedlot performance, carcass traits, and rumen morphometric parameters of Nellore cattle would be observed when ITM were replaced with reduced OTM, in proteinate forms, either alone or in combination with exogenous amylase. The supplement inclusion remained constant across treatments to isolate the effects of trace mineral and exogenous amylase inclusion.

**Table 3.** Effects on feedlot performance and carcass traits in feedlot cattle fed reduced levels of organic trace minerals replacing inorganic trace mineral sources in combination with exogenous amylase.

Trace mineral source	Inorganic		Organic		SEM	*P* value		
Exogenous amylase inclusion	With	Without	With	Without		*T* ^a^	*A* ^b^	*T* × *A*
***Feedlot performance***								
** Body weight, kg**								
** Initial**	349.18	349.19	349.41	349.04	11.37	0.97	0.89	0.88
** Final**	564.67	562.59	572.93	570.38	13.42	0.11	0.64	0.96
** Average daily gain, kg**	1.94	1.92	2.01	1.99	0.04	0.07	0.60	0.99
** DMI^c^, kg/day**	11.44	10.99	11.47	11.09	0.34	0.72	0.03	0.85
** DMI, % of BW**	2.51	2.43	2.50	2.44	0.03	0.99	0.02	0.72
** G: F^d^ ratio, kg/kg**	0.171	0.175	0.176	0.180	0.004	0.06	0.10	0.97
***Carcass traits***								
** Hot carcass weight, kg**	315.07	313.15	318.51	317.64	8.27	0.18	0.63	0.86
** Dressing percentage, %**	55.77	55.64	55.58	55.69	0.26	0.78	0.95	0.63
** Ultrasound data**								
** 12th-rib fat, mm**								
** Initial**	2.23	2.31	2.21	2.27	0.06	0.56	0.18	0.86
** Final**	6.02	5.98	5.97	6.48	0.25	0.38	0.37	0.29
** Biceps femoris fat thickness, mm**								
** Initial**	2.23	2.31	2.21	2.27	0.06	0.56	0.18	0.86
** Final**	9.20	9.01	10.18	9.63	0.40	0.05	0.36	0.65
** Longissimus muscle area, cm^b^**								
** Initial**	50.30	50.66	50.38	50.73	1.38	0.94	0.73	0.99
** Final**	88.12	89.13	89.04	90.35	1.32	0.28	0.24	0.88
** Marbling, %**								
** Initial**	2.09	2.28	2.34	2.29	0.08	0.15	0.43	0.18
** Final**	2.51	2.52	2.66	2.60	0.10	0.28	0.82	0.75

a Trace mineral source effect.

b Exogenous amylase inclusion effect.

c Dry matter intake.

d Gain-to-feed.

Initial and final BW did not differ among treatment groups (*P ≥* 0.11). The DMI, when expressed in kg/d or as a percentage of BW, was also unaffected by trace mineral source (*P ≥* 0.72). However, cattle fed OTM tended to show higher ADG (*P = *0.07) and G: F ratio (*P = *0.06) compared with those fed ITM. Although Dorton et al. (2006) and Ryan et al. (2015) suggested that OTM sources may improve bioavailability and metabolic utilization, particularly under conditions that promote oxidative or metabolic stress, and Suttle (2010) proposed that such responses may reflect enhanced mineral absorption and incorporation into key enzymes involved in energy and antioxidant metabolism, the present data should not be interpreted as direct evidence of improved mineral bioavailability. No mineral status biomarkers (e.g., plasma or hepatic trace mineral concentrations) were collected in this study. As a result, the performance trends observed should be interpreted as physiological outcomes associated with feeding OTM under the conditions of this trial, rather than as direct evidence of enhanced mineral absorption

It is important to understand that this experiment was conducted under commercial feedlot conditions, with a focus on growth performance outcomes. For this reason, blood or liver sampling was not included in the experimental design. Although such measurements would have provided more direct insight into mineral bioavailability, logistical constraints prevented these sample collections. We recognize this as a limitation and recommend that future research include mineral status indicators to clarify the mechanistic basis underlying potential differences between OTM and ITM sources. In contrast, Kegley et al. (2012) and Lippolis et al. (2017) did not report the effect of organic complexed or inorganic cobalt, copper, manganese, and zinc supplementation on ADG and G: F ratio in shipping-stressed beef cattle or feeder cattle supplemented during a 45-day preconditioning period, which was not the case in this study.

It is important to highlight that all cattle in this study were bulls and did not receive anabolic implants or β-agonists. Genther-Schroeder et al. (2016) and Samuelson et al. (2016) indicate that trace mineral supplementation, particularly Zn, may interact with these technologies to influence muscle growth and carcass traits. Under implanted or β-agonist-fed conditions, mineral requirements often increase, producing stronger responses to supplemental trace minerals. In the present study, mineral requirements may have been lower, potentially contributing to the modest OTM response. Future studies should evaluate whether reduced OTM supplementation levels produce different outcomes in implanted or β-agonist-fed cattle.

Overall, the results of this study are consistent with previous research evaluating OTM and ITM supplementation in feedlot cattle. Caldera et al. (2017) reported limited differences in performance when OTM replaced ITM sources. In contrast, Guimaraes et al. (2022) and Lippy et al. (2022) observed improvements in G: F ratio or mineral status under conditions of greater physiological demand. Loh et al. (2025) also demonstrated that OTM may influence rumen epithelial characteristics and metabolic markers, although responses remain inconsistent across studies. Dorton et al. (2006) and Heldt and Davis (2019) reported that the effects of trace mineral form often depend on the stress level of the animals, the presence of growth-promoting technologies, and interactions with dietary starch content. The literature demonstrates that OTM effects in feedlot cattle are context-dependent and may be more pronounced when mineral requirements are elevated by stress, disease pressure, implants, or β-agonist conditions, which are not present in the current study.

Cattle fed diets supplemented with exogenous amylase in this study had greater DMI, whether expressed in kg/d (11.5 *vs*. 11.0 kg/d; SEM: 0.31; *P = *0.03) or as a percentage of BW (2.51 *vs*. 2.43%; SEM: 0.02; *P = *0.02), without improvements (*P ≥* 0.10) in ADG or G: F ratio compared to cattle fed diets without the exogenous amylase. Although exogenous amylase may enhance ruminal starch hydrolysis and increase the availability of fermentable substrates (Meschiatti et al. 2019), several factors may account for the absence of a growth response. The starch concentration of the finishing diet (∼30% of DM) was moderate compared with diets in which amylase supplementation typically elicits stronger performance effects (often exceeding 40% starch; Tricarico et al. 2007; Silva et al. 2023). Under such conditions, starch may not have been limiting, and additional enzymatic hydrolysis may not have meaningfully increased energy supply. Furthermore, improved starch digestibility does not necessarily enhance performance if post-ruminal nutrient use or microbial efficiency becomes limiting.

As described by DiLorenzo et al. (2011), even when exogenous amylase increases starch disappearance, cattle may not gain additional weight if the resulting energy is redirected toward maintenance processes rather than tissue deposition. Finally, the lack of a G: F response suggests that cattle may have compensated for increased DMI by altering ruminal fermentation dynamics or shifting nutrient partitioning toward maintenance rather than growth. Due to the ruminal pH, short-chain fatty acids profiles, and microbial protein synthesis not measured in this study, the precise mechanisms underlying this response remain unclear. However, it is plausible that the additional energy consumed was used inefficiently and, therefore, insufficient to promote measurable improvements in growth.

There was no main effect of trace mineral form (*P ≥* 0.15) on HCW, dressing percentage, or any of the ultrasound-derived carcass measurements, including initial and final 12th-rib fat thickness, initial Biceps femoris fat thickness, initial and final longissimus muscle area, and initial and final marbling scores. However, cattle fed OTM had greater final Biceps femoris fat thickness (OTM: 9.91 mm *vs*. ITM: 9.11 mm; SEM: 0.29; *P* = 0.05) compared to those fed ITM sources, which may suggest that OTM may promote improved lipid metabolism and deposition, potentially through enhanced enzymatic activity in lipid synthesis pathways and better mineral status within adipose tissue (Spears 2003), even though this study did not assess lipid metabolism. Although no effect on 12th-rib fat deposition or marbling was observed in the present study, the increased Biceps femoris fat thickness may have practical implications for improving carcass quality, which are important economic traits in the beef cattle industry.

No main effect of exogenous amylase inclusion (*P ≥* 0.18) was observed for any of the carcass characteristics variables evaluated. Tricarico et al. (2007) reported greater HCW and longissimus muscle area with supplemental exogenous amylase in heifers fed both cracked and high-moisture corn finishing diets. The fact that experimental diets in this study contained about 30% starch may have limited the effectiveness of exogenous amylase (Silvestre et al. 2022), but further research on adding exogenous amylase to finishing diets with more starch should be conducted to explore if the amylase may have an application when different types of diets are fed.

Rumenitis score, number of papillae, mean papillae area, absorptive surface area, and papillae area did not differ between trace mineral sources or with exogenous amylase inclusion (*P ≥* 0.14; Table 4). No liver abscesses were observed in any of the animals evaluated in this study, indicating that neither mineral source nor exogenous amylase inclusion negatively affected rumen integrity or health. Nutritional challenges from feeding high-concentrate diets, like in this study, may cause digestive disorders and trigger systemic inflammation (Nagaraja and Titgemeyer 2007). However, no acute-phase proteins or inflammatory markers were measured in this study, and rumenitis scores were not different across treatments. To our knowledge, the relative significance of OTM on rumen morphometrics has not been evaluated. Heinrichs et al. (2007) indicated that amylase supplementation may stimulate rumen tissue development when incorporated into calf starter, with a dose-dependent, but not linear. In the current study, the lack of interaction effects suggests that mineral source and exogenous amylase act through distinct physiological pathways, without evidence of synergistic or antagonistic effects on ruminal morphology.

**Table 4.** Effects on rumen morphology in feedlot cattle fed reduced levels of organic trace minerals replacing inorganic trace mineral sources in combination with exogenous amylase.

Trace mineral source	Inorganic		Organic		SEM	*P* value		
Exogenous amylase inclusion	With	Without	With	Without		*T* ^a^	*A* ^b^	*T* × *A*
**Rumenitis score**	2.43	2.19	2.21	2.63	0.23	0.65	0.71	0.16
**Number of papillae, *n***	59.20	53.73	51.21	47.45	4.62	0.14	0.33	0.85
**Mean papillae area, cm2**	0.68	0.63	0.66	0.73	0.04	0.28	0.78	0.12
**ASA^c^, cm^2^/cm^2^ of rumen wall**	40.79	32.66	33.00	34.53	3.16	0.36	0.31	0.14
**Papillae area, % of ASA**	97.18	96.94	96.96	96.76	0.23	0.33	0.28	0.90

a Trace mineral source effect.

b Exogenous amylase inclusion effect.

c Absorptive surface area.

The tendency for greater ADG and G: F when feeding OTM sources suggests potential benefits for feed efficiency in finishing cattle. Given the variability reported across studies evaluating OTM responses, future research should examine the mechanisms of mineral bioavailability, metabolic enzyme activity, rumen function, and oxidative status. Although the present study showed a consistent increase in DMI with exogenous amylase inclusion, previous research has reported variable growth responses to exogenous amylase supplementation (Tricarico et al. 2007; Pech-Cervantes et al. 2022; Simon et al. 2024). Additional research is warranted to determine how factors such as grain processing, diet composition, and cattle genotype influence starch digestibility, microbial fermentation patterns, and overall feedlot performance, particularly under tropical conditions.

## Conclusion

Overall, replacing ITM with reduced levels of proteinate-based OTM, while maintaining selenium-yeast inclusion, did not exert adverse effects on growth performance, carcass characteristics, or rumen morphometrics of finishing Nellore bulls. Instead, OTM sources modestly improved ADG, G: F ratio, and Biceps femoris fat deposition, indicating that lower OTM supplementation rates can effectively support finishing cattle. Exogenous amylase supplementation for feedlot Nellore bulls in moderate-starch finishing diets may not translate into benefits for growth performance, carcass traits, or rumen morphometrics. The present data show that reduced OTM inclusion can replace traditional inorganic sources without compromising animal productivity or rumen health, offering a potentially more efficient mineral supplementation strategy for commercial feedlots.

## Abbreviations:

ADG: average daily gain
BW: body weight
DM: dry matter
DMI: dry matter intake
G: F: gain-to-feed
HCW: hot carcass weight
ITM: inorganic trace minerals
OTM: organic trace minerals
